# Cortical inexcitability in ALS: correlating a clinical phenotype

**DOI:** 10.1136/jnnp-2024-333928

**Published:** 2024-08-13

**Authors:** Nathan Pavey, Andrew Hannaford, Mana Higashihara, Mehdi van den Bos, Nimeshan Geevasinga, Steve Vucic, Parvathi Menon

**Affiliations:** 1 The University of Sydney, Sydney, New South Wales, Australia; 2 Department of Neurology, Concord Repatriation General Hospital, Sydney, New South Wales, Australia; 3 Department of Neurology, Tokyo Metropolitan Geriatric Hospital and Institute of Gerontology, Itabashi-ku, Tokyo, Japan; 4 Westmead Hospital, Westmead, New South Wales, Australia

**Keywords:** MOTOR NEURON DISEASE, NEUROPHYSIOLOGY, MOTOR

## Abstract

**Background:**

Cortical inexcitability, a less studied feature of upper motor neuron (UMN) dysfunction in amyotrophic lateral sclerosis (ALS), was identified in a large cross-sectional cohort of ALS patients and their demographic and clinical characteristics were contrasted with normal or hyperexcitable ALS cohorts to assess the impact of cortical inexcitability on ALS phenotype and survival.

**Methods:**

Threshold-tracking transcranial magnetic stimulation (TMS) technique with measurement of mean short interval intracortical inhibition (SICI) differentiated ALS patients into three groups (1) inexcitable (no TMS response at maximal stimulator output in the setting of preserved lower motor neuron (LMN) function), (2) hyperexcitable (SICI≤5.5%) and (3) normal cortical excitability (SICI>5.5%). Clinical phenotyping and neurophysiological assessment of LMN function were undertaken, and survival was recorded in the entire cohort.

**Results:**

417 ALS patients were recruited, of whom 26.4% exhibited cortical inexcitability. Cortical inexcitability was associated with a younger age of disease onset (p<0.05), advanced Awaji criteria (p<0.01) and Kings stage (p<0.01) scores. Additionally, patients with cortical inexcitability had higher UMN score (p<0.01), lower revised ALS Functional Rating Scale score (p<0.01) and reduced upper limb strength score (MRC UL, p<0.01). Patient survival (p=0.398) was comparable across the groups, despite lower riluzole use in the cortical inexcitability patient group (p<0.05).

**Conclusion:**

The present study established that cortical inexcitability was associated with a phenotype characterised by prominent UMN signs, greater motor and functional decline, and a younger age of onset. The present findings inform patient management and could improve patient stratification in clinical trials.

**WHAT IS ALREADY KNOWN ON THIS TOPIC**

- Cortical inexcitability is excluded from analysis in transcranial magnetic stimulation (TMS) studies assessing cortical dysfunction in amyotrophic lateral sclerosis (ALS) due to the absence of a measurable TMS parameter.

**WHAT THIS STUDY ADDS**

- This study assesses cortical inexcitability in ALS patients and contrasts their demographic and clinical characteristics to those with cortical hyperexcitability or normal cortical inhibition to better understand the patient phenotype associated with cortical inexcitability, its implication for patient management and stratification for clinical trials, and the insights it provides into ALS pathogenesis.

**HOW THIS STUDY MIGHT AFFECT RESEARCH, PRACTICE OR POLICY**

- This study identifies cortical inexcitability occurring in a substantial proportion of the ALS cohort and defines the clinical and demographic characteristics of the patient group with cortical inexcitability which would inform their management and recruitment into clinical trials. This study enhances insight into ALS pathogenesis and would progress neurophysiological studies and complement pathological and imaging research in ALS.

## Introduction

 Amyotrophic lateral sclerosis (ALS) is a disease of the motor system with upper and lower motor neuron involvement.^1 2^ The assessment of upper motor neuron (UMN) dysfunction in ALS is aided by several investigational techniques^3^ including the well-described technique of transcranial magnetic stimulation (TMS).^4^,^6^ The most consistent cortical change reported in ALS is the phenomenon of hyperexcitability,^6 7^ which may aid the diagnosis of ALS with 73.2% sensitivity and 80.9% specificity at an early stage in the disease^8 9^ and may be an indicator of prognosis.^10^

Cortical inexcitability characterised by the absence of a motor-evoked response in a resting target muscle at maximal stimulator output^11^ is also a feature of cortical dysfunction in ALS.^9 12^ Cortical inexcitability was noted to be greater over motor cortical representations of body regions other than the hand^13^ and was associated with ALS disease progression,^12^ pronounced UMN signs^14^ and also linked with adverse prognosis.^15^

Dysfunction or degeneration of cortical neuronal circuits could account for cortical inexcitability in ALS, including the direct and transsynaptic excitatory circuits^16^ which are activated by TMS. Dysfunction of fast-conducting corticomotorneurons would also result in cortical inexcitability consequent to absent or desynchronised excitatory inputs onto spinal motor neurons connecting with the target muscle.^17^ Peripheral nerve measurements excluding markedly reduced or absent target muscle motor response would help exclude spinal motor neuron dysfunction causing high and unrecordable motor threshold on TMS testing^11^ thereby selecting a cohort of ALS patients with cortical causes of inexcitability for assessment.

Neuropathological studies in sporadic ALS associated with TDP-43 pathology^18^ suggest a stage-wise progression of pathology in the pyramidal cells and their axons and in other non-motor cortical regions which may underlie ALS phenotypes and disease progression. Neuroimaging in ALS reports precentral gyrus thinning in ALS specifically in faster progressing patient phenotypes and those with greater clinical UMN involvement.^19^ Corticospinal tract dysfunction measured by decreased fractional anisotropy and increased mean diffusivity was also described specifically with greater disease severity and faster disease progression.^20^ These pathophysiological mechanisms underlie ALS phenotypes and their contribution to cortical inexcitability phenotype would provide further insights into ALS pathophysiology.

The relevance of cortical inexcitability in ALS and its associated clinical phenotype remains to be fully elucidated. The aim of the present study was to address these key questions in a cross-sectional observational study between ALS patients with inexcitability, hyperexcitability or normal excitability of the motor cortex selected from a large well-phenotyped ALS cohort.

## Methods

ALS patients, diagnosed by the Awaji criteria^21^ who underwent threshold tracking TMS testing^22^ between January 2010 and June 2023, were included in the study. The TMS technique of threshold tracking used^22^ remained unchanged throughout the period and stimulation was performed using either a circular or figure of eight coil. Clinical data recorded at the time of TMS testing were collated for the following clinical variables: site of disease onset (limb, bulbar, respiratory, generalised), handedness,^23^ revised ALS Functional Rating Scale (ALSFRS-R)^24^ (maximum score 48), MRC muscle strength scores^25^ for the upper limb (shoulder abduction, elbow flexion, elbow extension, wrist extension, finger abduction and thumb abduction on both sides), (maximum score 60) and lower limbs (hip flexion, knee extension, foot dorsiflexion on both sides), (maximum score 30) along with MRC sum score (maximum score 90) and UMN score^26^ totalling pathologically brisk biceps, triceps, brachioradialis, finger, knee and ankle tendon reflexes on each side. Additional points were scored for a brisk jaw jerk and extensor plantar response on each side.

Lower motor neuron assessment was performed by stimulating the median nerve at the wrist and recording the compound muscle action potential (CMAP) response from the thenar abductor pollicis brevis (APB) muscle according to a previously described technique.^27^ Peak-to-peak CMAP amplitude (mV), distal motor latency (ms) and F-wave latencies (ms) were recorded. Patients with a peak-to-peak CMAP response <2 mV were classified as having a wasted thenar eminence. The neurophysiological index for the median nerve^28^ and the split hand index were calculated^29^ on both sides using previously reported formulae.

Threshold tracking TMS was performed on all ALS patients using the previously described technique.^22^ Specifically, the motor-evoked potential (MEP) amplitude was fixed and changes in test stimulus intensity required to generate a target response of 0.2 mV (±20%), when preceded by a subthreshold conditioning stimulus, were measured.^30^ Resting motor threshold (RMT) was defined as the stimulus intensity required to maintain the target MEP response of 0.2 mV (±20). Short interval intracortical inhibition (SICI) was recorded over the following interstimulus intervals (ISIs): 1, 1.5, 2, 2.5, 3, 3.5, 4, 5 and 7 ms. Stimuli were delivered sequentially as a series of three channels: channel 1: stimulus intensity, or threshold (% maximum stimulator output, %MSO) required to produce the unconditioned test response (ie, RMT); channel 2: response to the subthreshold conditioning stimulus (70% RMT); channel 3 records the stimulus (% maximal stimulator output) required to produce the target MEP following a subthreshold conditioning stimulus equal in intensity to 70% of RMT. Motor-evoked response was recorded over the APB muscle in the thenar eminence previously described^13^ to have preserved motor-evoked responses for a longer duration than lower limb and cranial muscles.

Based on cortical excitability testing, three ALS cohorts were identified; ‘inexcitable’, ‘hyperexcitable’ and ‘normal excitability’. Cortical inexcitability was defined as an absent MEP response with TMS intensity set to 95% of %MSO when stimulating one or both motor cortices. ALS patients with cortical hyperexcitability were defined by reduced mean SICI between ISIs of 1–7 ms (≤5.5%) in one or both motor cortices.^8 9^ Normal cortical excitability was defined as mean SICI (ISI 1–7 ms) >5.5%.

### Statistical analysis

Differences in demographic, clinical and neurophysiological variables were compared across the three ALS groups. Student’s t-test was used to compare means between paired groups, χ^2^ test, analysis of variance with post hoc correction (Tukey) or Kruskal-Wallis test were used for multiple comparisons. Kaplan-Meier curve was used to assess differences in survival across the three ALS groups. A p<0.05 was deemed clinically significant. Data are reported as mean±SD or median (IQR).

## Results

### Clinical phenotype

417 patients with ALS (242 men, mean age 61±13 years, 90% right and 10% left-handed) were included in the study. 71% of patients exhibited limb onset, 28% bulbar onset and 1% respiratory or generalised onset ALS. The median disease duration was 11 (7–19.5) months, median ALSFRS-R score was 41 (38–44), median upper limb strength score was 56 (50–60) and the median MRC sum score (sum of upper and lower limb strength) was 82 (76–87). Fifty-two percent of patients met the Awaji definite/probable diagnostic category with greater clinical disease dissemination while 48% were classified as Awaji possible or negative. Some ALS patients with flail arm, flail leg or bulbar-onset phenotype did not have a combination of upper and lower motor neuron signs in a body region and were Awaji negative on initial review but met diagnostic criteria on clinical follow-up. Of relevance, 57.5% of patients classified as Awaji possible met the Gold Coast diagnostic criteria with clinical signs of upper and lower motor neuron involvement in one body region. 36% of patients had commenced riluzole therapy at the time of assessment. Mean survival in the cohort was 2.3±2 years.

**Figure 1:**
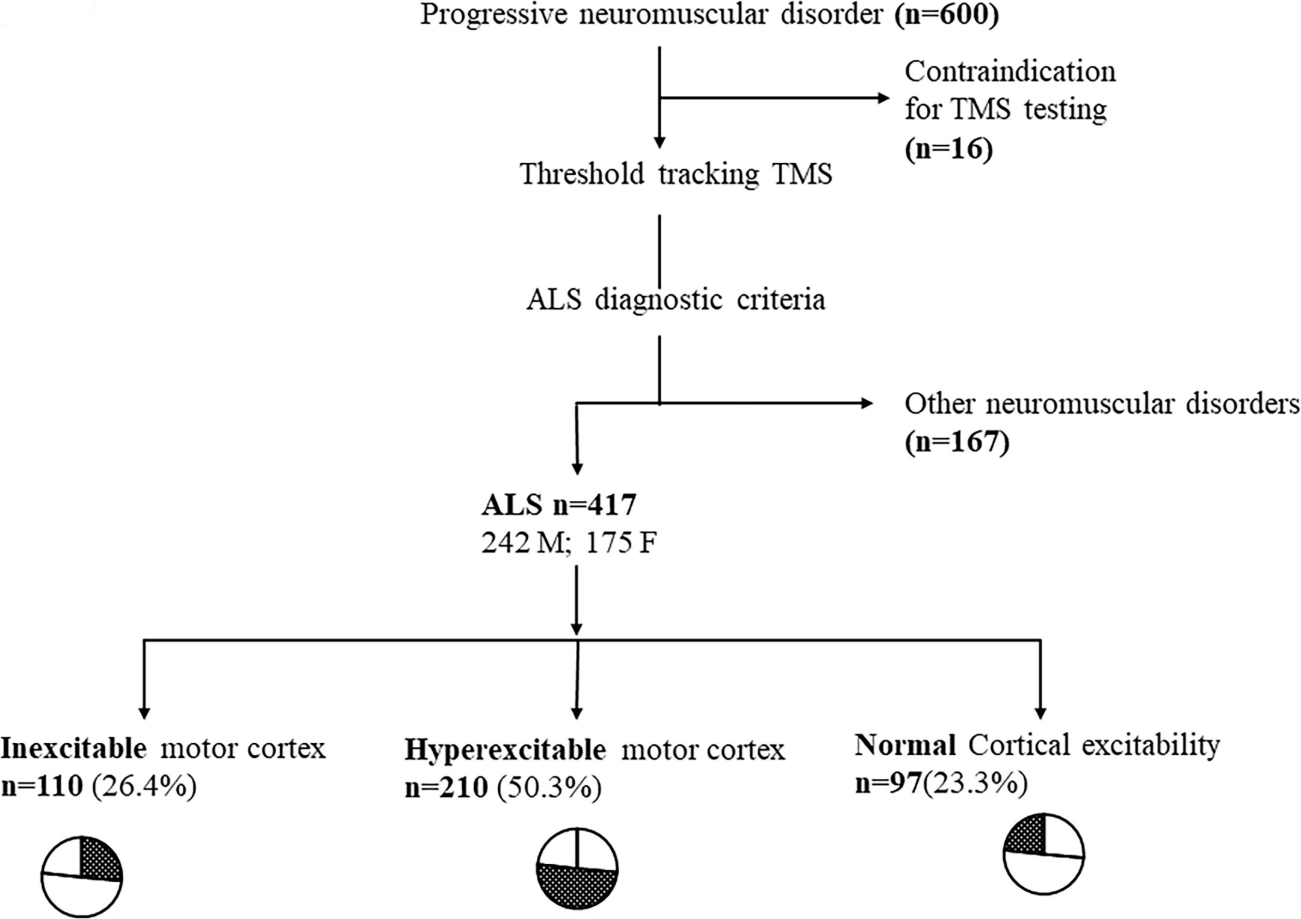
417 patients with ALS were identified from a large cohort who underwent transcranial magnetic stimulation studies. Patients were further classified as having an inexcitable, hyperexcitable or normal motor cortical response. Cortical inexcitability formed 26.4% of the entire cohort. ALS, amyotrophic lateral sclerosis; TMS, transcranial magnetic stimulation.

**Figure 2:**
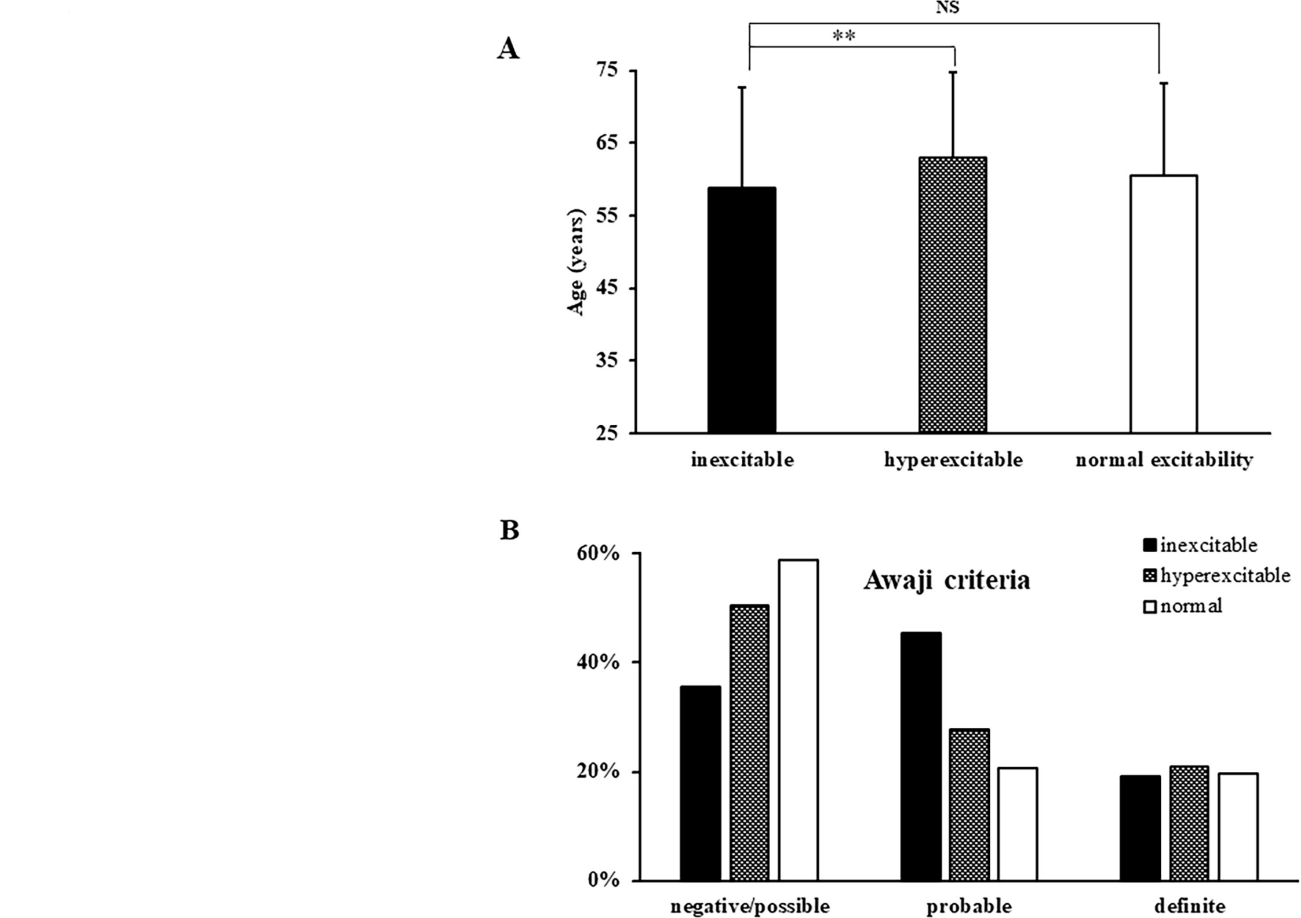
(A) Patients with cortical inexcitability were significantly younger when compared with patients exhibiting cortical hyperexcitability. (B) A higher percentage of patients with cortical inexcitability had Awaji probable and definite disease.

Cortical inexcitability was evident in 110 (26.4%), hyperexcitability in 210 (50.3%) and normal excitability in 97 (23.3%) ALS patients (figure 1). Of the patients with an inexcitable cortex, 5 (1.2%) had an eventual classification of primary lateral sclerosis (PLS) and were excluded from analysis. ALS patients exhibiting cortical inexcitability (58.2±13.8 years) had a younger age of disease onset when compared with patients with cortical hyperexcitability (62.9±11.8 years, df=312, t=−3.1, p=0.002) and comparable with those exhibiting normal cortical excitability (60.6±12.6 years, df=200, t=−1.27, p=0.206; figure 2A; table 1). A greater proportion of ALS patients exhibiting cortical inexcitability were classified as Awaji probable/definite (65.7%, p<0.001, figure 2B, table 2) when compared with ALS patients exhibiting cortical hyperexcitability (49%) and normal excitability (40.6%).

**Table 1:** Patients with cortical hyperexcitability had significantly higher upper motor neuron scores,lower ALSFRS-R scores, and reduced upper limb strength (MRC upper limbs) than the other groups. They were significantly younger than the cortical hyperexcitability group. Lower limb strength (MRC lower limbs) and disease duration at the time of testing were comparable between the three groups

Variable	Inexcitable cortex mean±SD median (IQR)	Hyperexcitable mean±SD median (IQR) (pairwise significance)	Normal excitability mean±SD median (IQR) (pairwise significance)
Age (years)	58.2±13.8	62.9±11.8 (p=0.002)	60.6±12.6 (p=0.206)
UMN score (max 15)	12 (6)	11(6) (p=0.012)	11 (8) (p=0.011)
ALSFRS-R (max 48)	39 (7)	41(6) (p=0.010)	42 (4) (p=0.002)
MRC upper limb (max 60)	54 (12)	58 (7) (p<0.001)	58 (10) (p=0.004)
MRC lower limbs (max 30)	28 (4)	29 (4) (p=0.976)	28 (8) (p=0.051)
Disease duration (months)	17.9±17.2	15.4±15.6 (p=0.640)	13.6±13.5 (p=0.196)

ALSFRS-R, revised Amyotrophic Lateral Sclerosis Functional Rating Scale; MRC, Medical Research Council Score; UMN, Upper Motor Neuron.

**Table 2:** Patients with cortical inexcitability had advanced disease stage by the Awaji diagnostic category and King’s staging criteria. Fewer patients with cortical inexcitability were on riluzole at the time of testing suggesting diagnostic delay. A greater proportion of patients had limb rather than bulbar onset disease across all groups

	Inexcitable N (%)	Hyperexcitable N (%)	Normal N (%)	df	P value, phi
Awaji category					
Negative/possible	36 (34.3)	106 (51)	57 (59.4)	4	<0.001, 0.217
Probable	49 (46.7)	58 (27.9)	20 (20.8)		
Definite	20 (19.0)	44 (21.1)	19 (19.8)		
Kings stage					
1	26 (25)	68 (48.9)	40 (58.0)	8	0.001, 0.289
2	57 (54.8)	47 (33.8)	20 (29.0)		
3	21 (20.2)	23 (16.6)	9 (13.0)		
4a	0 (0.0)	1 (0.7)	0 (0.0)		
Riluzole use					
No	83 (79.8)	115 (55.8)	60 (61.9)	4	<0.001, 0.206
Yes	21 (20.2)	91 (44.2)	37 (38.1)		
Site of onset					
Bulbar	25 (23.8)	66 (31.7)	25 (25.8)	6	0.449, 0.118
Limb	80 (76.2)	141 (67.8)	72 (74.2)		
Respiratory	0 (0.0)	1 (0.5)	0 (0.0)		

**Figure 3:**
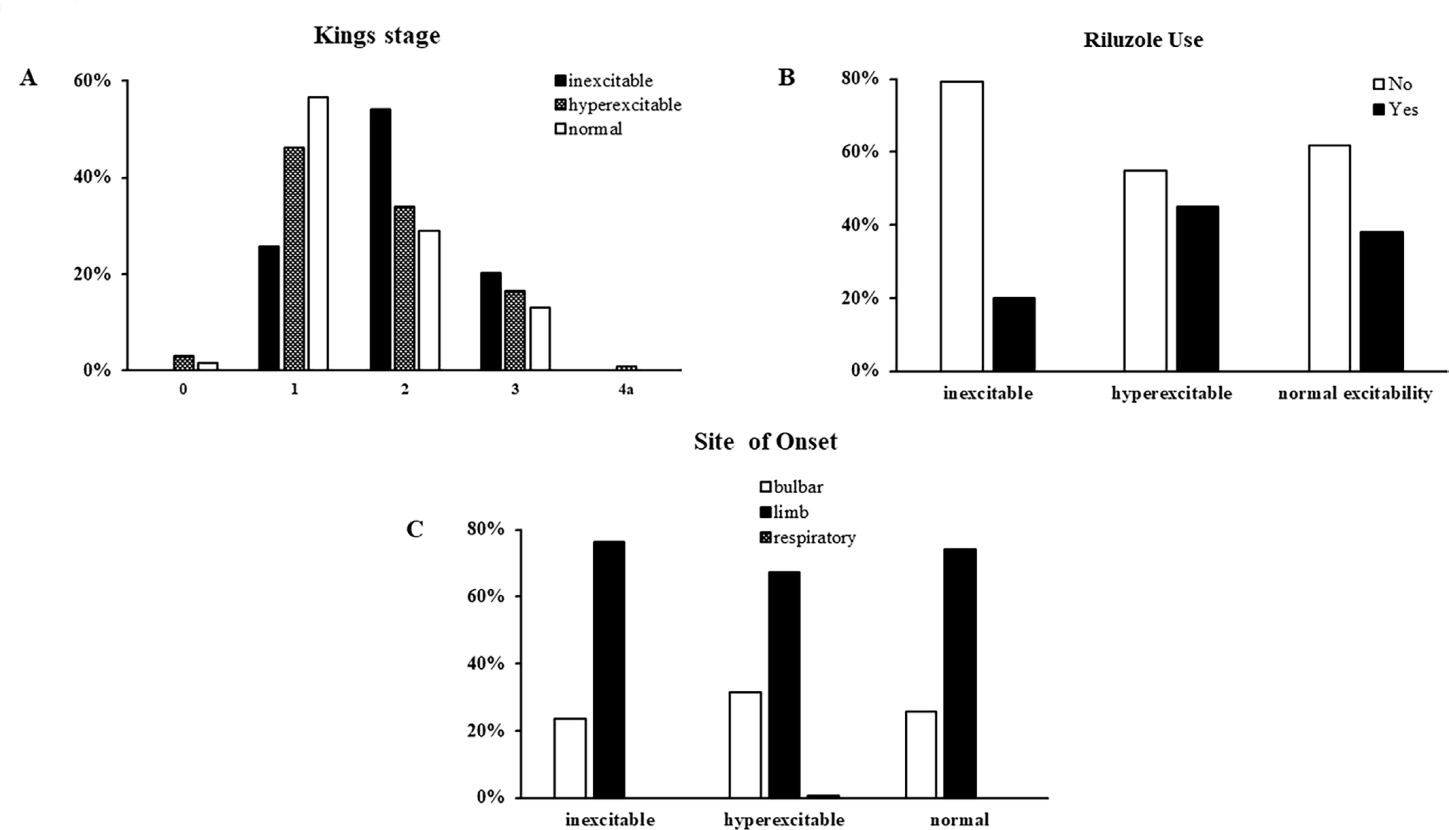
(A) Patients with cortical inexcitability had advanced clinical disease as defined by the King’s staging criteria. (B) Riluzole use indicative of a definite diagnosis of ALS was significantly lower in patients with cortical inexcitability. (C) However, a similar proportion of patients in all three groups had bulbar and limb onset disease. ALS, amyotrophic lateral sclerosis.

Of further relevance, patients with an inexcitable cortex exhibited higher King’s staging scores of 2 and 3 denoting greater clinical disease spread (75%) (p=0.001, figure 3A, table 2) when compared with ALS patients exhibiting cortical hyperexcitability (50.4%) or normal excitability (42%). Riluzole was used less frequently in patients with cortical inexcitability (p<0.001, figure 3B, table 2). In contrast, the proportion of patients with limb and bulbar-onset disease was comparable across the three cohorts (p=0.449, figure 3C, table 2).

**Figure 4:**
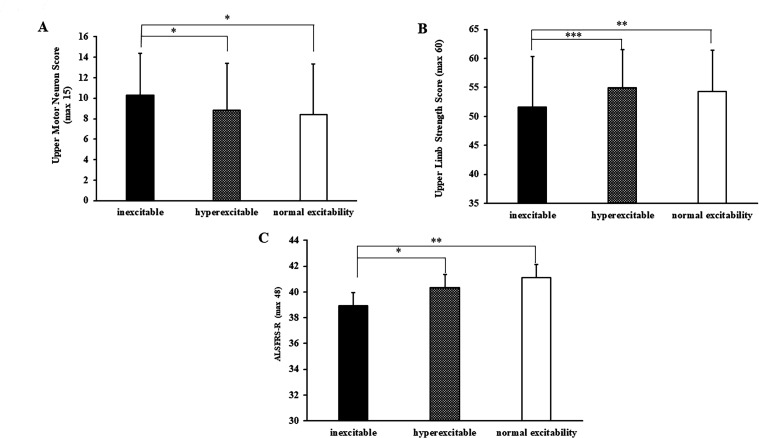
(A) Patients with cortical inexcitability had significantly higher upper motor neuron scores when compared with patients with cortical hyperexcitability and normal cortical excitability. (B) Patients with cortical hyperexcitability had reduced upper limb strength score. (C) The ALSFRS-R functional score was significantly lower in patients with cortical inexcitability when compared with patients exhibiting hyper and normal cortical excitability. ALSFRS-R, revised Amyotrophic Lateral Sclerosis Functional Rating Scale.

Separately, the UMN score was significantly higher in ALS patients with cortical inexcitability (median 12, IQR 6) compared with those exhibiting cortical hyperexcitability (median 11, IQR 6; p=0.012) or normal cortical excitability (median 11, IQR 8; p=0.011, figure 4A). There was prominent upper limb weakness in patients with an inexcitable motor cortex as evidenced by significantly smaller upper limb MRC scores (ALS _INEXCITBALE_ 54 (12), ALS _HYPEREXCITBALE_ 58 (7), p<0.001; ALS _NORMAL CORTICAL FUNCTION_ 58 (10), p=0.004, figure 4B). Greater functional disability was also evident in patients with cortical inexcitability as indicated by a significantly smaller ALSFRS-R score (ALS _INEXCITBALE_ 39 (7), ALS _HYPEREXCITBALE_ 41 (6), p=0.010; ALS _NORMAL CORTICAL FUNCTION_ 42 (4), p=0.002; figure 4C). In contrast, there were no significant differences across the groups in lower limb muscle strength and disease duration (table 1). Moreover, survival was comparable across the three cohorts (log rank χ^2^=1.8, df=2, p=0.398).

### Neurophysiology assessment

Neurophysiology assessment for differences in lower motor neuron dysfunction across the three cohorts revealed a significant reduction in the split hand index in patients exhibiting cortical inexcitability, although this reduction was only significant on the right side (ALS _INEXCITBALE_ 4.0, ALS _HYPEREXCITBALE_ 6.1, p=0.008; ALS _NORMAL CORTICAL FUNCTION_ 6.1, p=0.023). In contrast, the peak-to-peak CMAP amplitude, recorded over the thenar eminence, as well as the neurophysiological index and split hand index on the left side were comparable across the three ALS cohorts (table 3).

**Table 3:** Split-hand index was reduced on the right side in patients with cortical inexcitability. The abductor pollicis brevis (APB) muscle compound muscle action potential (CMAP) and neurophysiological index were comparable across the groups on both sides

Variable (units)	Side	Inexcitable cortex Mean±SD	Hyperexcitable cortexMean±SD (pairwise significance)	Normal cortical excitabilityMean±SD (pairwise significance)
Split-hand Index	Right	4.0±3.8	6.1±4.0 (p=0.008)	6.1±3.7 (p=0.023)
	Left	3.9±3.1	5.1±3.8 (p=0.113)	4.9±4.1 (p=0.585)
APB CMAP (mV)	Right	6.1±4.0	7.0±3.8 (p=0.37)	7.0±4.1 (p=0.78)
Left	7.0±4.1	7.8±4.3 (p=0.91)	6.6±4.3 (p=1.0)
Neurophysiological index	Right	1.2±1.3	1.4±1.6 (p=1.0)	1.5±2.0 (p=0.91)
	Left	1.1±0.9	1.4±1.1 (p=0.20)	1.3±1.1 (p=1.0)

APB, abductor pollicis brevis; CMAP, compound muscle action potential.

## Discussion

The present study established that cortical inexcitability was associated with a specific ALS phenotype, characterised by younger age of onset, greater functional decline, more prominent UMN signs and greater upper limb weakness. The clinical phenotype was also accompanied by a greater reduction of the split hand index in the right hand, the dominant hand in the majority of the cohort. Interestingly, survival, disease duration at the time of testing and site of disease onset were comparable across the three cortical subgroups. The pathophysiological mechanisms underlying the associations and clinical relevance of the phenotype are further discussed.

RMT measures the integrated excitability across the motor cortical system and gives a functional evaluation of the pyramidal system by reflecting the ease with which corticomotoneurons are excited.^11^ In ALS, RMT is reduced in early stages of the disease, preceding the onset of muscle wasting^11^ and may be associated with profuse fasciculation and hyper-reflexia.^31^ With disease evolution, there is a progressive increase in RMT eventually leading to cortical inexcitability.^31^ At a pathophysiological level, the initial reduction in RMT is indicative of cortical and spinal motor neuron hyperexcitability.^11^ As the disease progresses, progressive dysfunction or degeneration of corticomotorneurons, and/or the interneuronal circuits that connect with them, occurs leading to increased motor thresholds and eventually cortical inexcitability.^31^

An increase in motor thresholds and cortical inexcitability has been reported in atypical ALS phenotypes, most notably PLS.^32^ The present cortical inexcitability cohort exhibited prominent clinical UMN signs, like that observed in PLS. However, their younger age of onset, greater degree of function decline and more advanced disease course, as indicated by the Awaji^21^ and King’s stages, suggest similarities with the predominantly UMN ALS phenotype.^33^

Of relevance, previous studies report the finding of an inexcitable motor cortex in a minority (~10%) of typical patients at their first clinical presentation.^15^ The present larger cohort revealed a sizeable proportion (26.4%) of patients exhibiting cortical inexcitability on presentation. The presence of cortical hyperexcitability across all four limbs within the first year of symptom onset was also reported to be a biomarker of adverse prognosis and a faster rate of disease progression.^15^ Survival in the present inexcitable cohort, however, was comparable to the hyperexcitable and normal excitability groups. The discordant findings between studies may relate to assessing patients at differing clinical disease stages, underpinned by clinical and pathological heterogeneity and variable rates of disease progression. Alternately, the younger age of the cortical inexcitability cohort may confer a survival advantage overriding their advanced disease stage and greater functional disability.^34^

Of further relevance, cortical inexcitability was associated with a greater degree of upper limb weakness, particularly the split hand phenomenon^35^ on the dominant right side. Given that the upper limb has a greater cortical representation within the motor cortex,^36^ and that RMTs reflect the density of corticomotoneuronal projections onto spinal motor neurons as well as the excitability of large motor cortical neurons (Betz cells), the present findings could be explained on an anatomical basis.^11^ Specifically, a greater degree of neurodegeneration within the motor cortex (Betz cells) and corticospinal tracts may underlie cortical inexcitability resulting in predominant signs in the upper limbs especially on the dominant side.

It has been argued that motor neuronal degeneration is mediated by cortical hyperexcitability,^37^ a hypothesis seemingly discordant with the present findings, the hyperexcitable phase may precede presentation in the current inexcitable ALS cohort due to the rapidity of progression of neuronal dysfunction. Further, riluzole therapy was less frequently used in ALS cohort with cortical inexcitability. As initial TMS testing may be used as a diagnostic tool in ALS, the reduced use of riluzole could reflect a diagnostic delay caused by the atypical phenotype of the cortical inexcitability cohort. Given that riluzole modulates cortical excitability, it could be argued that the present findings may have been influenced by the less frequent riluzole use in the cortical inexcitability ALS cohort. This seems unlikely as riluzole therapy was shown to partially normalise SICI, for a transient period of ~3 months but not modify RMT.^38 39^

### Relevance to clinical management and trials

The finding of distinct cortical signatures across the ALS cohort while significant in understanding ALS pathophysiology is also of relevance to patient management and recruitment into clinical trials. Specifically, the presence of cortical inexcitability appears to suggest a specific ALS phenotype, with a younger age of onset, more prominent functional decline and a greater degree of upper limb weakness. Consequently, management in a multidisciplinary clinic should address these specific functional deficiencies to improve quality of life. From a clinical trials perspective, the presence of cortical inexcitability may be important in patient stratification that could in turn impact trial outcomes. Importantly, TMS parameters have been used as outcome biomarkers in ALS,^40^ enabling assessment of target engagement and efficacy. Incorporation of the TMS technique into clinical trial designs would help further clarify its utility in patient stratification.

## Data Availability

The data that supports the findings of this study is available from the corresponding author, on reasonable request.
